# Age-related patterns of early childhood development practices amongst rural families in Burkina Faso: findings from a nationwide survey of mothers of children aged 0-3 years

**DOI:** 10.1080/16549716.2020.1772560

**Published:** 2020-06-30

**Authors:** Jennifer Hollowell, Mireille Belem, Tessa Swigart, Joanna Murray, Zelee Hill

**Affiliations:** a Development Media International, London, UK; b Development Media International, Ouagadougou, Burkina Faso; c Department of Epidemiology and Public Health, Institute for Global Health, University College London, London, UK

**Keywords:** Burkina Faso, early childhood development, parenting, survey research, rural population

## Abstract

**Background:**

Around two thirds of children in Sub-Saharan Africa are at risk of not meeting their developmental potential. Scalable interventions, based on an understanding of local contexts, that promote nurturing care in children’s early years are needed.

**Objectives:**

To investigate age-related patterns of Early Childhood Development (ECD) practices amongst caretakers of children aged 0–3 years in rural households in Burkina Faso, in order to inform the design of a mass media campaign to be evaluated through a randomized controlled trial.

**Methods:**

A household survey using a structured questionnaire was used to collect data from 960 rural mothers of children aged 0–3 years in a regionally stratified random sample of 130 villages.

**Results:**

The mother was the main caretaker and engaged most in ECD-related activities at all ages (0–3 years). The father, grandmother and older children also engaged in ECD-related activities with older children (aged 1–3 years). Singing and playing occurred moderately frequently. Singing in the last three days: 36% at age 0–5 months increasing to 84% at age 3 years; playing in the last three days: 26% at age 0–5 months, increasing to 65% at age 3 years. Activities such as reading, counting, drawing, ‘showing and naming’ and ‘chatting’ were limited, particularly in the child’s first year. Reasons for not engaging in these activities include lack of literacy, lack of books and toys or playthings and a belief that the child was too young.

**Conclusion:**

Opportunities for learning, especially through verbal interactions, appeared to be limited during the developmentally crucial first three years, most notably in the first year of life. The challenge for ECD intervention development in Burkina Faso will be finding ways to promote more responsive interactions at an early age and finding ways of mobilizing other family members to become more engaged in stimulating activities in the child’s early years.

## Background

Globally, around 250 million children under five years of age are at risk of not reaching their developmental potential. Some of the highest levels are found in Sub-Saharan Africa (SSA) where 66% of children under five are at risk of not reaching their potential, compared with 43% globally [1]. Sub-optimal development in the early years has long-term consequences for the health and wellbeing of the child, as well as negative economic and developmental implications for the country [2,3]. The WHO Nurturing Care Framework [4] was launched in 2018 to provide a ‘roadmap for action’ to stimulate investment in early childhood development, which the WHO argues is a highly cost-effective way of boosting a country’s prosperity, expanding equitable opportunities and ending extreme poverty. The report emphasizes the need for countries to assess the current situation in order to inform local initiatives and program planning, but the evidence on ECD practices from low- and middle-income countries (LMICs) is extremely limited. Available data indicate relatively low levels of adult support for early learning and limited access to books and playthings in countries in SSA compared with other LMICs [5,6,7,8]. However, the evidence relates predominantly to children aged 3–4 years [5,6] or aged 0–5 years [8], with only one study examining patterns specifically in younger children (infants aged 0–11 months [7]). There appears to be no published quantitative data from SSA on patterns of stimulation across the crucially important first three years of life.

There is strong evidence that parent-focused, face-to-face interventions promoting nurturing care and stimulation can improve child development outcomes [9], and can compensate for developmental delays due to risks such as poverty and malnutrition [9,10,11]. However, such interventions can be challenging and expensive to deliver at scale [12,13].

The survey findings reported here form part of a mixed-methods research programme in Burkina Faso to support the development of a mass media campaign targeting parenting behaviours, which will be evaluated through a randomized controlled trial. A mass media approach could be a more easily scalable alternative to face to face interventions, with the role of media in changing social norms around childhood, creating awareness and promoting best practice recognized [4]. Such an approach (using radio, which has good penetration in rural Burkina Faso) has already been demonstrated to be an effective means of changing health-related behaviours [14,15]. The aim of this survey was to quantitatively investigate current ECD practices amongst parents and other caretakers of children up to three years old in rural households in Burkina Faso, in order to inform the design of the mass media campaign. Our particular focus was on describing the prevalence, and age-related patterns, of activities that stimulate children’s cognitive, language and socioemotional development; and on describing the patterns of involvement of key household members in these activities.

We also conducted a separate in-depth qualitative study using focus groups to explore childcare practices and caregivers’ perceptions of ECD-related practices in Burkina Faso, which is reported elsewhere [16]. Our focus in both studies was on rural areas because the population of Burkina Faso is predominantly rural (78% [17]) and evidence suggests that children in rural areas of Burkina Faso receive substantially less home stimulation than those in urban areas [18].

## Methods

The data for this study was collected by adding an ECD module to a questionnaire used in a rolling monthly household survey run by Development Media International (DMI). The survey, which started in June 2016, used a team of 7 trained interviewers to collect data from rural households.

### Instrument development

We developed the ECD module based on the ‘Family support for learning’ survey questions used in a UNICEF KAP survey in the Solomon Islands [19]. The ‘support for learning’ section of the questionnaire covered similar ECD activities to the UNICEF MICS survey instruments [19] but included additional questions and prompts which we felt were useful in our target population. We included all questions related to adults playing, telling stories, singing, reading/looking at pictures and drawing/counting with the child. Additionally, because we wanted to capture activities involving responsive interaction with the child, we added questions on ‘chatting’ and ‘showing and naming’ objects.

We were also interested in understanding who were the main caretakers of children other than mothers. We therefore added questions about whether the mother left the child with others when she went out, how frequently this occurred, and who cared for the child in her absence. We did not conduct formal pretesting, but interviewers conducted trial interviews with women as part of their training and feedback was used to make minor modifications to these questions where needed.

### Data source – the DMI household survey

The DMI monthly household survey was originally designed to monitor DMI’s radio campaigns (on family planning and child survival). It was conducted in a regionally stratified random sample of small rural villages (less than 5000 inhabitants) situated in the transmission zones of 14 radio stations that broadcast DMI campaigns. All eligible villages (n = 2606) within the transmission zones were identified and grouped into 7 geographical regions to create a sampling frame of villages. Within each zone, a list of the villages, in random order, was created and each month interviews were conducted in the next 7 villages on each of the zone lists (6 during the rainy season when access to villages can be more difficult). If the interviewers were unable to conduct interviews in a selected village for any reason during the allocated month (e.g. no access due to flooding or security concerns) they replaced that village with the next one on the list.

Within each selected village, the aim was to interview the husband and wife in ten households: eight with children aged less than 5 years, and two recently married couples without children. In polygamous households only one wife (the mother of the youngest child in the household) was interviewed.

There were no readily available sampling frames at village level (household listings) to enable households to be randomly sampled so the interviewers identified households by approaching the village chief or village committee to obtain a list of twenty ‘broadly representative’ eligible households within the village. Ten households were then approached, and consent obtained prior to conducting the interviews. Where either husband or wife did not consent, the couple was replaced by another on the list.

Although at a village level the sample was not randomly selected, an evaluation comparing the data with routinely collected sociodemographic data for rural Burkina Faso (J Townend, personal communication) suggests that the survey sample was broadly representative in terms of religion, ethnicity, languages spoken, and household items such as mosquito nets. The proportion lacking any education was marginally higher than the average for rural men and women of reproductive age. Since only one wife was interviewed per household, women in polygamous marriages were underrepresented.

### Study participants for the ECD study

As in the MICS, we collected data on ECD practices by interviewing the mother. Women who participated in the DMI household survey were eligible for the ECD module if the interview took place during the study period (1 April – 30 June 2018) and the woman had a child aged 0–3 living with her at the time of the interview.

### Data collection

The questionnaire was programmed into a Personal Digital assistant (PDA) in French using survey CTO software [20]. The new ECD module was introduced to the survey interviewers at a one-day training session. The questionnaire was written in French but delivered in the respondents’ local languages (Dioula, Mooré, Dagara, Nouni, Kasséna, Goulmanchéma, Fulfuldé).

### Statistical analysis

Weighted percentages were calculated using the Stata/SE (Version 15) survey command, using inverse probability weights based on each village’s probability of selection and the probability of selection of an individual within the village (based on the total population of the village as reported in the 2006 national census). Although the sample was not random within villages, for the purposes of calculating confidence intervals we treated the sample as a stratified, clustered random sample with villages as the primary sampling unit, and used robust standard errors to account for clustering.

## Results

Interviews were conducted in 130 villages. Twelve villages (9%) in the original sample had to be replaced, 6 because of security concerns in the areas, four because the villages were inaccessible (generally due to flooding) and two because funerals were being held in the village in the week of the interviewer’s visit.

The questionnaire was administered to 962 mothers of children aged 0–3 years (inclusive). Two eligible mothers did not respond to the ECD questions for unknown reasons and were excluded, leaving an analysis sample of 960.

Mothers’ characteristics are described in Table 1. The mothers ranged in age from 17 to 47 with a median age of 29. Just under one third were in polygamous marriages with two thirds of these women not being the husband’s first wife. The vast majority of mothers (78%) had no education and were not literate. Around half of the mothers (47%) had engaged in some form of income generating activity in the previous two weeks, most commonly selling food.

**Table 1.** Mother, child and household characteristics.

	n	Weighted % (95% CI)	
Mother’s age			
Median (IQR)	29 (25–33)		
<21	109	10.9	(8.3–14.3)
22–28	327	36.1	(31.4–41.0)
29–36	415	42.3	(37.4–47.4)
37–48	109	10.7	(8.4–13.5)
Marital status			
Married–monogamous	671	67.2	(62.3–71.8)
Married–polygamous	289	32.8	(28.2–37.7)
Number of births			
1	96	9.9	(7.1–13.7)
2–3	317	34.0	(30.1–38.1)
4–6	442	45.6	(40.4–50.9)
5–11	104	10.5	(6.8–15.9)
Youngest child’s age			
0–5 months	146	17.9	(13.8–22.9)
6–11 months	157	17.5	(13.9–21.9)
1 year	255	25.3	(21.0 – 30.2)
2 years	272	27.9	(24.8–31.2)
3 years	130	11.4	(9.2–14.0)
Child’s sex			
Male	468	50.7	(46.1–55.3)
Female	492	49.3	(44.7–53.9)
Mother’s education			
No education	780	78.2	(72.8–82.8)
Some education or literate	180	21.8	(17.2–27.2)
Mother’s economic activity			
Economically inactive	494	52.8	(47.2–58.3)
Economically active	466	47.2	(41.7–52.8)
Number of children under 5 in household*			
1	325	33.2	(27.6–39.4)
2	453	49.1	(44.6–53.5)
3	121	13.0	(10.3–16.2)
4–6	61	4.7	(3.3–6.8)
Total number of children in household*			
1	100	9.8	(6.9–13.8)
2–3	247	25.3	(22.4–28.4)
4–6	362	38.2	(33.9–42.7)
≥7	251	26.6	(22.3–31.4)
All	960	100	

* Includes all children in the household

Around one third of households contained only one child aged under 5, but most also contained older children and only 10% contained only one child. The age of the mother’s youngest (index) child ranged from less than one month to three years. As the survey asked about the mother’s youngest child, the distribution of ages was slightly skewed towards younger ages, with over half (61%) of the children aged 0–1 years.

### Caretakers other than the mother during the day

Around 90% of mothers with a child aged <1 year reported that they never left the child with someone else when they went out during the day. By age 3 years, the proportion left with someone else most days had increased to 48% (Supplementary Table S1). Information about how long children were left with others was limited, with most mothers saying ‘it depends’.

For the 15% of mothers who left a child aged 0–3 years with someone else most days, the woman’s mother or mother-in-law was the most frequent caretaker (44%), followed by children aged less than 15 (30%). Other adults were less frequently mentioned as caretakers (2–7%).

### ECD–related behaviours

The most commonly mentioned ECD–related behaviours reported to have been carried out by an adult in the household within the last 3 days were playing (57%) and singing (63%). Telling stories (16%), ‘chatting’ with the child’ (14%) and ‘showing and naming objects’ (15%) were less commonly reported. Reading or looking at picture books (3%), counting or drawing objects (5%) were only reported in a small minority of households (Table 2).

**Table 2.** ECD related behaviours in the past 3 days.

		Child’s age						
		0–5 mo	6–11 mo	1 yr	2 yrs	3 yrs	Total	(95% CI)
*In the last 3 days, did an adult in the household:*								
Tell stories to the child								
	n	3	10	24	66	65	168	
	Weighted %	1.3	4.5	9.0	25.1	51.7	16.2	(12.3–20.9)
Sing songs to the child								
	n	46	91	148	186	103	574	
	Weighted %	35.8	61.3	61.7	73.3	84.0	62.8	(57.3–68.0)
Count or draw objects with child								
	n	2	2	7	29	25	65	
	Weighted %	0.4	0.6	0.9	7.8	18.9	4.8	(3.4–6.6)
Read or look at picture books with the child								
	n	1	1	5	11	9	27	
	Weighted %	0.3	0.3	1.4	4.9	5.8	2.5	(1.4–4.3)
	missing (n)			1				
Play with the child								
	n	45	81	178	199	91	594	
	Weighted %	25.7	47.1	70.0	67.7	65.4	56.9	(49.4–64.1)
‘Chat’ with the child *								
	n	4	8	26	59	49	146	
	Weighted %	2.2	2.0	12.9	22.6	33.2	14.1	(10.8–18.2)
Show and name objects to child *								
	n	1	8	28	67	36	140	
	Weighted %	0.3	5.1	14.2	26.1	24.1	14.6	(11.0 – 19.0)
Take the child out of the house**								
	n	25	34	65	78	30	232	
	Weighted %	59.6	75.5	81.7	92.0	77.7	79.6	(68.9–87.3)
	N	146	157	255	272	130	960	

* New questions (not in UNICEF survey)

** Question added after start of survey, data collected for one month only (N = 293)

Overall, 80% of children had been taken out of the house by an adult in the past 3 days, most commonly by the mother, sometimes by the father, particularly at older ages, and less frequently by a child aged 15 and over.

All of these activities were carried out more frequently with older children (Table 2), and ECD- related behaviors for children aged 0–5 months were particularly low. For some behaviors the change with age was marginal, with very low levels even for older children (counting or drawing objects, reading or looking at books, showing and naming objects). For others the difference was more marked, for example, only 9% of children one year of age had a story told to them in the last 3 days compared to 52% of three-year olds.

### Playing

In households where an adult played with the child, mothers appeared to play most frequently with the child (76%), particularly at younger ages (96% at age 0–5 months decreasing to 55% at age 3 years). Children aged 15 and over (41%), fathers (29%) and, to a lesser extent, grandmothers (16%) also played with the child in some households (Table 3), although this was uncommon for children aged 0–5 months.

**Table 3.** Who played with the child?

		Child’s age						
		0–5 mo	6–11 mo	1 yr	2 yrs	3 yrs	Total	(95% CI)
Mother	n	43	69	139	138	52	441	
	Weighted %	96.3	86.3	78.4	71.1	54.6	75.5	(69.2–80.8)
Father	n	8	19	57	60	36	180	
	Weighted %	8.1	22.1	34.9	28.4	34.1	28.6	(23.6–34.2)
Child aged 15+	n	6	22	86	95	45	254	
	Weighted %	14.8	24.8	47.3	45.4	48.2	40.9	(33.9–48.4)
Grandmother	n	7	16	21	30	16	90	
	Weighted %	14.2	22.7	15.3	14.9	17.3	16.4	(11.0–23.8)
Grandfather	n	0	1	3	3	4	11	
	Weighted %	0.0	0.8	2.3	1.7	2.2	1.7	(0.8–3.4)
Other adult	n	0	2	5	1	0	8	
	Weighted %	0.0	4.2	4.0	0.5	0.0	2.0	(1.0–4.2)
Total	N	45	81	178	199	91	594	

Multiple responses possible: Percentages do not sum to 100%

In households where the mother had played with the child in the past three days, mothers reported that the children played with toys bought in a shop or market (38%), ‘objects from outside’ e.g. sticks and stones (39%), everyday objects (bowls, plates, cups, etc.) (32%), toys made at home (23%) and objects that play music (17%). Less than 1% of children played with objects for drawing and writing (Supplementary Table S2).

In households where no adult played with the child (43%), the child being too young was the most frequent reason (82% on average but with a marked age-related trend: 100% at age 0–5 months decreasing to 38% at age 3 years). No access to toys or playthings was another relatively common reason (31%). ‘Not important for children’ was mentioned as the reason in 8% of households, more commonly for older children aged 2 and 3 years (Supplementary Table S5).

### Singing

In the 63% of households where the mother reported that an adult sang to the child, the mother herself was the person most likely to sing to the child (86%). Fathers (23%), older children aged 15 and over (21%) and grandmothers (16%) also sang to the child in some households (Table 4). Children’s songs (64%) and/or songs in the mother’s own language (80%) were the most commonly mentioned (Supplementary Table S3).

**Table 4.** Who sang to the child?

		Child’s age						
		0–5 mo	6–11 mo	1 yr	2 yrs	3 yrs	Total	(95% CI)
Mother	n	43	84	130	141	75	473	
	Weighted %	93.1	95.7	89.2	80.7	74.6	85.7	(80.9–89.5)
Father	n	5	20	39	41	34	139	
	Weighted %	11.6	21.2	26.3	19.8	37.2	23.4	(17.5–30.7)
Child aged 15+	n	5	9	42	51	36	143	
	Weighted %	15.9	8.4	26.1	21.6	31.0	21.3	(16.2–27.5)
Grandmother	n	7	17	18	34	19	95	
	Weighted %	12.8	15.2	13.2	19.3	17.5	16.2	(11.9–21.6)
Grandfather	n	2	2	4	1	1	10	
	Weighted %	5.1	0.7	2.8	0.3	0.1	1.45	(0.5–4.1)
Other adult	n	1	2	3	3	0	9	
	Weighted %	1.7	1.7	1.5	2.9	0.0	1.77	(0.9–3.5)
Total	N	46	91	148	186	103	574	

Multiple responses possible: Percentages do not sum to 100%

The most common times when the mother reported singing to the child were while taking care of the child during the day (71%), while preparing food or doing household tasks (36 %) and while giving the child a bath (24%). Singing to the child while breastfeeding (9%) or when putting the child to bed (6%) were mentioned less frequently (Supplementary Table S4).

### Chatting with the child

An adult in the household chatted with the child ‘about what you are doing or things that interest the child’ infrequently (14%).

### Reasons for not reading to the child or looking at picture books with the child

In households where no adult read or looked at picture books with the child (97%), the stated reasons for not reading to the child or looking at picture books were: parents cannot read (75%), the child is too young (58%) and no access to books or reading material (25%). Over 85% of children aged less than one year were considered to be ‘too young’ to be read to or to look at picture books, dropping to 26% at age 3 (Table 5). As the majority of villagers were not literate, the question was found to cause offence to some respondents and was amended in the third month of the survey to asking why an adult did not read ‘or look at picture books’, but this had a negligible effect on the pattern of responses.

**Table 5.** Reason for not reading to the child.

		Child’s age							
		0–5 mo	6–11 mo	1 yr	2 yrs	3 yrs	Total		(95% CI)
Why did an adult not read to child in the last three days?*									
No access to books or reading material									
	n	28	49	81	93	41	287		
	Weighted %	14.2	25.3	25.2	29.4	26.7	24.5		(18.8–31.3)
Parents too busy/working									
	n	2	1	6	8	5	22		
	Weighted %	0.5	0.5	2.5	1.9	5.4	1.9		(1.0–3.6)
Parents cannot read									
	n	76	107	216	234	112	745		
	Weighted %	41.3	60.0	87.1	89.4	93.0	75.1		(69.1–80.3)
Child is too young									
	n	115	124	127	88	23	477		
	Weighted %	88.7	87.1	50.0	36.6	26.5	57.7		(51.1–64.1)
Total	N	145	156	249	261	121	932		

*Question amended in month 3 to why parent did not ‘read or look at picture books’.

Multiple responses possible: Percentages do not sum to 100%.

## Discussion

The purpose of this study was to investigate current caregiver ECD practices among rural households in Burkina Faso, as part of a programme of research to help inform the development of a mass media campaign to promote behaviours supporting early childhood development. Findings show that the mother was the main caretaker of children aged 0–3 years, but grandmothers play an increasing caregiver role with older babies and children. Other adult members of the household, particularly the father and older children, also increasingly engaged in ECD-related activities with older babies and children. Activities such as singing and playing, were carried out moderately frequently, particularly among children aged over 5 months, while activities such as reading, counting, drawing, ‘showing and naming’ and ‘chatting’ were generally limited, particularly in the child’s first one to two years. Reported reasons for not engaging in these activities include lack of literacy amongst adults in the household, lack of books and toys or playthings and, particularly for babies and younger children, that the child was too young. Most of the ECD-related activities were carried out markedly less frequently in the child’s first two years than at ages 2–3 years.

The child’s first three years are regarded as the most crucial for early brain development [10,21,22] but most available data on ‘adult support for early learning’ in SSA relate to children older than this (either 3–4 years [5,6], or to a broad age range 0–5 years [8]). One multi-country study using data from the third round of the MICS (which included Burkina Faso), examined patterns of activities in the child’s first 12 months and found broadly increasing trends with age. The study found that increasing proportions of caregivers read, told stories, and named, counted, and drew with each additional month of the infant’s life; while the proportions of caregivers who played, sang songs, and took their infants outside increased at first but then levelled off at around 4–5 months [7]. Our findings are generally consistent with an age-related upward trend found by Bornstein, although our data additionally suggest that this continues beyond 12 months. The evidence thus suggests a large gap in caregiver practices at a crucial time for brain development. Some practices, such as storytelling and singing, need to be transitioned to lower age groups whilst others, such as counting or drawing and showing objects, need to be introduced across the ages. The messages in the mass media campaign may need to be different for these two types of behaviours.

It is also notable that the MICS data for infants aged 0–11 months in Burkina Faso in 2006 suggest low levels of key ECD-related activities compared with the average levels observed across West African countries with similar levels of development as measured by the United Nations Development Index [23]: 0% vs. 4% for reading to the infant in the last 3 days, 2% vs. 14% for telling stories, and 15% vs. 40% for naming, counting and drawing [8]. Comparable figures for all 38 LMICs in Bornstein’s analysis were 9%, 12% and 37% respectively [8]. We did not identify more recent data to determine how support for early learning in Burkina Faso currently compares with other counties in the region.

Our survey results support the findings of our separate, linked qualitative study [16], which was conducted using focus groups in two villages in central Burkina Faso. For example, the low levels of verbal interaction (telling stories, ‘chatting’ and showing and naming) that were found in this nationwide survey are consistent with the limited levels of interactive, verbal communication that were noted in our qualitative research with rural parents and grandmothers [16]. However, adults did sing to their children moderately frequently. Low levels of interaction have also been observed in a broadly similar qualitative study in Malawi [24]. As we have discussed elsewhere [16], caregivers in Burkina Faso may lack knowledge about the importance of responsive interaction and talking to young children from an early age [24]; and it does not appear to be the norm in rural African families to have conversations or communicate with young children [25,26].

Our survey findings suggest that the mother is the main caregiver in the child’s first year and that fathers have limited involvement in ECD activities even at older ages. However, findings from our linked qualitative study suggested that fathers (in the two villages where we conducted focus groups) were willing to engage in ECD activities and found it rewarding when they did so [16] suggesting that they are a potentially untapped ECD resource. Similarly, although they were generally not primary caregivers, our survey showed that grandmothers and older children in the household sang and played with young children. Our focus groups with grandmothers also indicated that they had more discretionary time than mothers to interact with their grandchildren, suggesting that they too can have a role to play in ECD.

A strength of our study is that data were collected from a relatively large sample of households in a stratified random sample of villages across rural Burkina Faso. Our findings provide an overview of age-related patterns of ECD-related practices in rural Burkina Faso, but there may well be regional variations which we did not explore. We were not able to randomly sample households within villages but an evaluation of respondents in the rolling household survey from which our sample was drawn has been found to be broadly representative of households in rural Burkina Faso (see ‘design of the survey’ above for details). The present survey was conducted during April to June, which is a predominantly dry season. Findings from our Focus Group study [16] suggest that adults tend to have markedly less free time to engage with their children during the rainy season (June/July to September), so our survey findings may not be generalizable to times of the year when adults are more heavily involved in agricultural work. We used a French-language questionnaire, based on a version of the MICS ECD questionnaire module [19] but because French is not widely spoken in rural areas the interviews were conducted in local languages (Dioula, Mooré, Dagara, Nouni, Kasséna, Goulmanchéma, Fulfuldé). Translation into local languages is considered best-practice but is not always feasible. On-sight translation is fairly widely used in countries such as Burkina Faso where there are multiple local languages and dialects, none of which are commonly used in written form [27]. The interviewers received face-to-face training on the new ECD module and carried out training interviews with women before going into the field. A limitation of our study is that we have no means of assessing whether translation issues during the interviews may have biased our findings.

As in the MICS, we asked questions about ’playing’ and about objects that the child played with, but qualitative evidence suggests that physical play, which parents believe helps their children to become stronger and healthier, may be common [24,26]. It seems conceivable that the MICS question on play objects may ‘prime’ respondents to focus on specific play activities when answering the play question. To limit this potential priming in our survey, the question about play objects was asked after the main playing question (in contrast to MICS). However, it remains unclear what activities play-related survey questions capture.

We included a question on ‘chatting’ because we felt that it was important to capture data on interactive verbal communication which is not covered by existing MICS questions, but a limitation is that the question that we used was pretested for comprehension but was not otherwise validated. We worded the question in a way that we considered had face validity (‘In the past three days have you chatted to [name] about what you are doing or about things that interest [name]’); we piloted the question to test for comprehension; and the concept being captured was discussed during interviewer training to ensure that it was correctly captured in translation to local languages. However, feedback from interviewers during fieldwork, which was followed up in our formative research, suggested that the concept of ‘chatting’ to children appears to have been baffling to some respondents (‘talk about what?’ was one response), while others appeared to be uncertain whether the question included chatting to children while playing. We would recommend further work to develop and validate questions to capture this aspect of cognitive stimulation in the early years.

## Conclusion

In rural Burkina Faso, the mother is generally the main caretaker and the adult who most commonly engages in ECD activities with children aged 0–3 years. The father, grandmother and older children also engage in ECD-related activities with older children (aged 1–3 years). Adults sing to older babies and children but other cognitively stimulating responsive verbal interaction with children in Burkina Faso appears to be limited during the developmentally crucial first three years, but most notably in the first year of life. Playing and singing appear to be more widespread although the nature of common play activities remains unclear. The challenge for ECD intervention development in Burkina Faso will be finding ways to promote more responsive verbal interaction at an early age and possibly finding ways of mobilizing other family members to become more engaged in stimulating activities in the child’s early years.

## Supplementary Material

Supplemental Material

## Data Availability

Data sharing requests should be addressed to the corresponding author.

## Paper context

Stimulation and responsive caregiving in the first three years of life are crucial for a child’s cognitive and socioemotional development and future prospects; while at a societal level, suboptimal development of children has negative implications for a country’s future prosperity and development potential. This study describes current patterns of early childhood development-related activities in rural families in Burkina Faso. The overall aim is to help inform the development of a mass media behaviour change campaign.
