# Supplementary material for: Age-related patterns of early childhood development practices amongst rural families in Burkina Faso: findings from a nationwide survey of mothers of children aged 0-3 years
Source: Glob Health Action. 2020 Jun 30;13(1):1772560. doi: 10.1080/16549716.2020.1772560 (PMC7480591; doi:10.1080/16549716.2020.1772560)
Supplement: Supplemental Material [file ZGHA_A_1772560_SM1668.docx]

**SUPPLEMENTARY FILE**

**Table S1: How often mother leaves child with someone else when she goes out**

|  |  | **Child's age** | | | | |  |  |
| --- | --- | --- | --- | --- | --- | --- | --- | --- |
|  |  | **0-5 mo** | **6-11 mo** | **1 yr** | **2 yrs** | **3yrs** | **Total** | **(95% CI)** |
|  |  |  |  |  |  |  |  |  |
| Every day or almost every day | n | 2 | 19 | 23 | 78 | 68 | 190 |  |
|  | Weighted % | 0.58 | 8.37 | 7.36 | 21.69 | 48.39 | 14.99 | (12.1 - 18.5) |
|  |  |  |  |  |  |  |  |  |
| 2-3 times a week | n | 3 | 1 | 61 | 95 | 34 | 194 |  |
|  | Weighted % | 2.52 | 0.21 | 28.06 | 37.18 | 32.36 | 21.65 | (16.9 - 27.3) |
|  |  |  |  |  |  |  |  |  |
| Once a week | n | 7 | 5 | 78 | 48 | 16 | 154 |  |
|  | Weighted % | 3.42 | 1.71 | 24.16 | 27.72 | 12.63 | 16.2 | (12.5 - 20.7) |
|  |  |  |  |  |  |  |  |  |
| *Never* | n | 134 | 132 | 93 | 51 | 12 | 422 |  |
|  | Weighted % | 93.47 | 89.71 | 40.42 | 13.41 | 6.62 | 47.17 | (42.0 - 52.4) |
|  |  |  |  |  |  |  |  |  |
| Total | N | 146 | 157 | 255 | 272 | 130 | 960 |  |

**Table S2: What objects does the child play with?**

|  | **Child’s age** | | | | |  |  |
| --- | --- | --- | --- | --- | --- | --- | --- |
|  | **0-5 mo** | **6-11 mo** | **1 yr** | **2 yrs** | **3yrs** | **Total** | **(95% CI)** |
| **Weighted percentages** | **%** | **%** | **%** | **%** | **%** | **%** |  |
| Toys bought in a shop or market | 50.1 | 47.1 | 33.9 | 40.4 | 15.9 | 38.1 | (31.3 - 45.4) |
| Toys made at home | 3.1 | 11.0 | 38.7 | 17.2 | 27.8 | 22.7 | (16.7 - 30.) |
| Objects that play music | 14.0 | 25.4 | 14.0 | 18.3 | 12.8 | 17.1 | (12.3 - 23.4) |
| Objects for drawing or writing | 0.0 | 0.0 | 0.0 | 0.0 | 1.2 | 0.1 | (0.0 - 0.9) |
| Everyday objects (bowls, plates, cups or pans)* | 5.2 | 34.4 | 25.5 | 45.6 | 38.2 | 32.4 | (25.9 - 39.6) |
| Objects from outside (sticks or stones) | 4.0 | 2.7 | 18.7 | 16.7 | 39.1 | 15.8 | (11.4 - 21.5) |
| None of the above | 19.2 | 7.3 | 4.7 | 0.0 | 1.0 | 4.8 | (2.3 - 9.6) |
| Total | 43 | 69 | 139 | 138 | 52 | 441 |  |
| *Categories not mutually exclusive*  *Denominator: Mother played with child in the last 3 days* | | | | | | | |
| * Most frequently mentioned everyday objects that child likes playing with (130 respondents): plates/dishes, boxes, bowls, cups. Occasionally mentioned: cans, tyres, natural object (sticks/stones/wood/sand), chalk. | | | | | | | |

**Table S3: What did the mother sing to the child?**

| **What did the mother sing to the child?** |  |  |  |
| --- | --- | --- | --- |
|  | **%** | **Weighted %** | **(95% CI)** |
| Pop songs or songs heard on the radio | 28.1 | 31.6 | (25.1 - 38.9) |
| Children's songs (“chansons enfantines”) | 64.3 | 63.8 | (55.9 - 71.1) |
| Songs in mother's own language | 82.9 | 79.9 | (73.7 - 84.9) |
| Songs to teach the child to count, colours, etc | 4.6 | 7.3 | (2.0 - 22.7) |
| *Not mutually exclusive, n=473* |  |  |  |

**Table S4: When did the mother sing to the child?**

|  | **%** | **Weighted %** | **(95% CI)** |
| --- | --- | --- | --- |
| When changing or dressing him | 8.9 | 6.6 | (4.0 - 10.7) |
| While giving him a bath | 22.8 | 23.9 | (17.5 - 31.8) |
| While preparing food or doing daily household tasks | 46.7 | 35.5 | (27.0 - 44.9) |
| At bedtime | 5.7 | 5.6 | (3.5 - 8.9) |
| While breastfeeding | 12.5 | 9.0 | (6.1 - 13.1) |
| While taking care of the child at home during the day | 62.6 | 70.5 | (63.3 - 76.8) |
| Some other time | 9.5 | 16.5 | (9.6 - 26.8) |
| *Not mutually exclusive, n=473* |  |  |  |

**Table S5: Reasons for not playing with the child**

|  |  | **Child's age** | | | | |  |  |
| --- | --- | --- | --- | --- | --- | --- | --- | --- |
|  |  | **0-5 mo** | **6-11 mo** | **1 yr** | **2 yrs** | **3yrs** | **Total** | **(95% CI)** |
| ***Why did an adult not play with the child in the last three days?*** | | | | | | |  |  |
|  |  |  |  |  |  |  |  |  |
| **No access to toys/playthings** | | |  |  |  |  |  |  |
|  | n | 13 | 18 | 31 | 31 | 20 | 113 |  |
|  | Weighted % | 5.89 | 10.84 | 26.24 | 30.83 | 42.29 | 19.08 | (12.1 - 28.8) |
| **Parents too busy/working** | | |  |  |  |  |  |  |
|  | n | 2 | 1 | 5 | 7 | 7 | 22 |  |
|  | Weighted % | 1.50 | 0.25 | 13.39 | 8.23 | 19.64 | 6.39 | (3.5 - 11.3) |
| **Child is too young** | |  |  |  |  |  |  |  |
|  | n | 101 | 73 | 62 | 50 | 15 | 301 |  |
|  | Weighted % | 100.00 | 98.26 | 79.11 | 67.58 | 52.81 | 84.86 | (78.1 - 89.8) |
| **Not important for children** | | |  |  |  |  |  |  |
|  | n | 0 | 3 | 8 | 11 | 8 | 30 |  |
|  | Weighted % | 0.00 | 3.11 | 4.23 | 14.21 | 23.64 | 6.54 | (3.8 - 11.2) |
| Total | N | 101 | 76 | 77 | 73 | 39 | 366 |  |
| Multiple responses possible: Percentages do not sum to 100% | | | | | | | | |
